# Vascular Endothelial Function Assessed by Flow-Mediated Vasodilatation in Young Adults Born Very Preterm or With Extremely Low Birthweight: A Regional Cohort Study

**DOI:** 10.3389/fped.2021.734082

**Published:** 2021-09-24

**Authors:** Britt Engan, Mette Engan, Gottfried Greve, Maria Vollsæter, Karl Ove Hufthammer, Elisabeth Leirgul

**Affiliations:** ^1^Department of Heart Disease, Haukeland University Hospital, Bergen, Norway; ^2^Department of Clinical Science, University of Bergen, Bergen, Norway; ^3^Department of Pediatric and Adolescent Medicine, Haukeland University Hospital, Bergen, Norway; ^4^Centre for Clinical Research, Haukeland University Hospital, Bergen, Norway

**Keywords:** endothelial function, flow-mediated dilatation, very preterm, extremely low birthweight, cardiovascular risk

## Abstract

**Background:** Preterm birth and low birthweight have been associated with increased risk of cardiovascular disease in young adults. Endothelial dysfunction is established as an early marker for development of atherosclerotic cardiovascular disease. Previous studies of endothelial function in young adults born very preterm or with extremely low birthweight have, however, shown diverging results.

**Objective:** We aimed to evaluate the risk of cardiovascular disease as measured by vascular endothelial function in young adults born very preterm (<29 weeks of gestation) or with extremely low birthweight (<1,000 g), compared with term-born controls.

**Methods:** This study included 50 young adults born very preterm or with extremely low birthweight and 49 term-born controls born in Norway in the periods 1982–1985, 1991–1992, and 1999–2000 at mean age 28 (±6) years. The endothelial function was assessed by ultrasound measured flow-mediated dilatation (FMD) of the right brachial artery. The arterial diameter was measured at baseline, after release of 5 min of occlusion, and after sublingual administration of nitroglycerine. FMD was reported as absolute and percentage diameter change from baseline and relative to nitroglycerine-induced dilatation.

**Results:** The participants were mainly normal weight non-smokers, without hypertension, diabetes, or established cardiovascular disease. The cases and controls had mean blood pressure 112/71 (SD 12/9) and 112/69 (SD 11/8) mmHg, body mass index 24.0 (SD 4.2) and 24.4 (SD 4.5) kg/m^2^, and HbA1c 32.7 (SD 2.5) and 33.0 (SD 2.6) mmol/mol, respectively. For both groups, 4 (8%) were smokers. Mean FMD for the adults born very preterm or with extremely low birthweight was 0.17 mm (95% CI 0.14, 0.21) vs. 0.24 mm (95% CI 0.20, 0.28) for the controls (*p* = 0.01), corresponding to a percentage increase of 5.4% (95% CI 4.2, 6.6) and 7.6% (95% CI 6.2, 8.9), respectively (*p* = 0.02). The FMD relative to maximal nitroglycerine-induced dilatation was 20% and 31%, respectively (*p* = 0.001).

**Conclusions:** Young adults born very preterm or with extremely low birthweight have significantly lower FMD compared with the term-born controls suggesting an increased risk of cardiovascular disease.

## Introduction

Preterm birth is an important cause of neonatal morbidity and mortality worldwide. In the 1970s, most neonates born extremely preterm (<28 weeks of gestation) in Norway did not survive. Technical and medical development in neonatal intensive care over the last decades have markedly increased survival rates for these neonates, which today exceed 80% (1–3). Neonates born extremely preterm now represent ~0.5% of all children growing up in Norway (4).

There is increasing evidence indicating that children born preterm (before 37 weeks of gestation) or with low birthweight (LBW) (<2,500 g) carry a risk of poor long-term health outcomes and a higher risk of young adult death, even in individuals born only moderately to late preterm (2, 5) (see Supplementary Table 1 for definitions of different degrees of preterm birth and low birthweight). Preterm birth and LBW have been linked to increased risk of hypertension (6, 7), diabetes (8, 9), and metabolic disease (9, 10), and it has been suggested that both preterm birth and LBW might constitute risk factors for later development of cardiovascular disease (5, 10–17). A recent meta-analysis concluded that LBW is associated with an overall increased risk of cardiovascular disease, coronary heart disease, and stroke (18). Furthermore, a population-based cohort study from the Nordic countries reported a 2-fold higher cardiovascular mortality in young adults born preterm in year 1967 to 2002 (2). The increased risk of all-cause mortality and cardiovascular disease are found to be inversely associated with gestational age (GA) and birthweight (2, 5).

A healthy endothelium is important for vascular homeostasis, and an appropriate balance of mediating factors regulates endothelial vasoconstriction and vasodilatation. A hallmark of healthy endothelial function is the nitric oxide synthesis and release by the endothelial inner lining of blood vessels in response to vasodilating stimuli (19). Vascular endothelial dysfunction constitutes an early step in the development of atherosclerosis and plays a role in the onset and progression of cardiovascular disease (19–22). Factors that adversely affect the endothelium include common cardiovascular risk factors such as age, hypertension, tobacco use, obesity, hyperlipidemia, insulin resistance, and physical inactivity (23). Endothelial dysfunction is also seen in patients with a family history of early cardiovascular disease but no other risk factors (23).

Previous studies of endothelial function in children and young adults born preterm or with LBW have presented diverging results, with findings of normal as well as reduced endothelial function (17, 24–28). Only a few studies have included young adults born extremely preterm or with extremely low birthweight (ELBW) (<1,000 g). However, Bassareo et al. (29) reported impaired microvascular endothelial function in young adults born with ELBW.

In the present study, we aimed to investigate how very preterm birth or ELBW may influence cardiovascular risk, by comparing the vascular endothelial function in young adults born at GA <29 weeks or with birthweight <1,000 g, with term-born controls.

## Materials and Methods

### Study Design and Participants

The Project Extreme Prematurity, organized by the WestPaed Research group in Bergen, Norway, have included three regional cohorts of young adults born very preterm at GA <29 weeks or with ELBW <1,000 g, from now on referred to as PB/ELBW. The three cohorts also included individually age- and sex-matched term-born controls, from now on referred to as controls. The participants have been followed longitudinally since year 2000, and have previously been examined in 2001 and 2008. The cohorts included a total number of 148 PB/ELBW and 138 controls. Subjects born in 1982–1985 (cohort 1) and in 1991–1992 (cohort 2) were retrospectively recruited, while those born in 1999–2000 (cohort 3) were prospectively recruited. The inclusion and follow-up of these cohorts have been described previously (30, 31).

**Figure 1:**
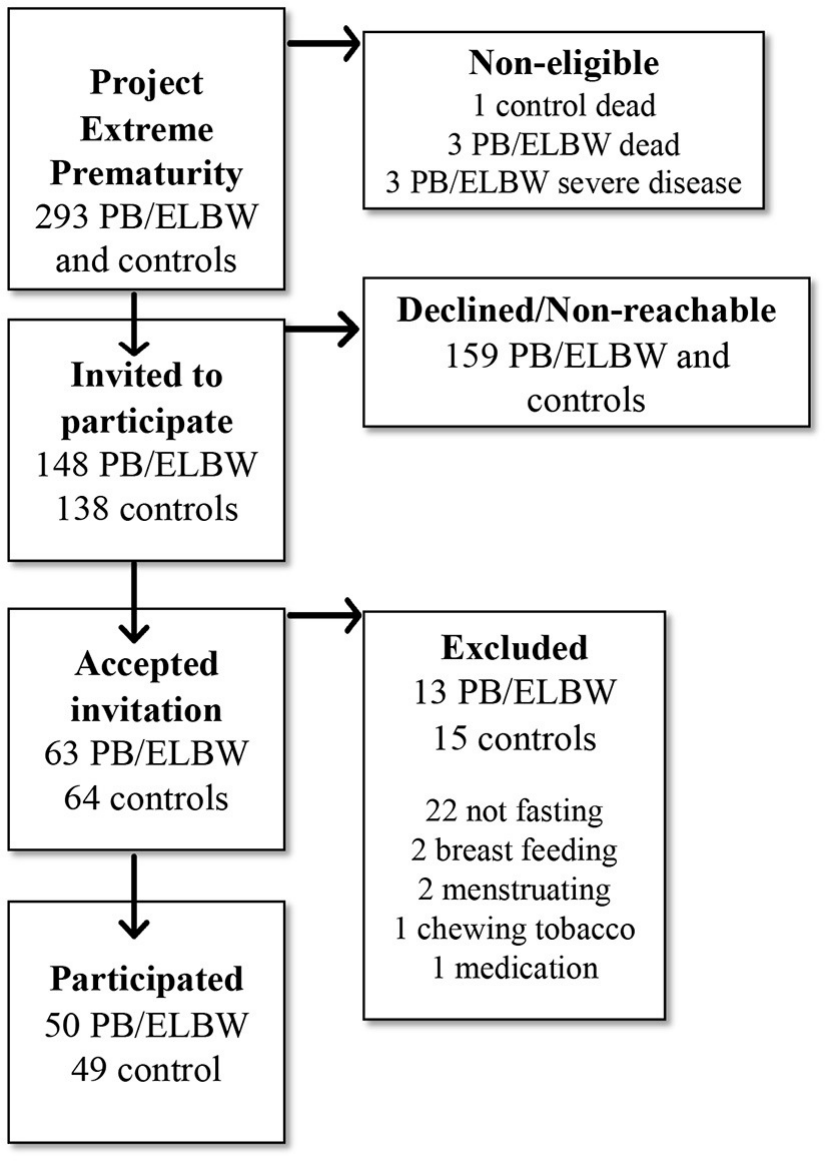
Flow chart of the study population in the study of vascular endothelial function in young adults born very preterm or with extremely low birthweight (PB/ELBW) and term-born controls, part of the Project Extreme Prematurity in Norway.

During the period, November 2017 to February 2020 individuals from the three regional cohorts were invited to a third follow-up, including a first-time non-invasive ultrasound-based examination of endothelial function. A total of 99 participants were examined, including 50 PB/ELBW (48% men) and 49 controls (41% men). Of the participants, 42 belonged to cohort 1, 29 to cohort 2, and 28 to cohort 3. HbA1c (glycosylated hemoglobin) was chosen as screening for diabetes. HbA1c is highly correlated with fasting blood glucose and established as a screening diagnostic test with cut-off for prediabetes 39 mmol/mol (32, 33). The analyses were performed in 39 (78%) of the PB/ELBW and in 47 (96%) of the controls. All participants went through a clinical examination including measurements of height, weight, and blood pressure. A flow diagram of the project participants is shown in Figure 1.

The Regional Committee for Medical and Health Research Ethics of the Western Norway Health Authority approved the study (REC 2017/0068). Written informed consent was obtained from all participants.

### Assessment of Endothelial Function by Flow-Mediated Dilatation of the Brachial Artery

Measurements of endothelial function by post-occlusive flow mediated dilatation (FMD) were performed according to guidelines by the International Brachial Artery Reactivity Task Force (34). Numerous factors affect flow-mediated vascular reactivity including the environmental temperature, ongoing infection, intake of food or drugs, and sex hormonal and sympathetic stimuli, among others (34). The assessment was therefore performed in supine position in a quiet, temperature-controlled room in the morning hours (before noon). Participant pre-test preparations included dietary restrictions (fasting for at least 8 h, and abstaining from caffeine and vitamin supplements on the examination day). In addition, all vasoactive medications and tobacco were withheld on the examination day, and the participants did not exercise the last 24 h before the examination. Participants who were pregnant, breast feeding, or menstruating were excluded to limit the variations in sex hormone levels in the female participants. Hormonal contraceptives have been shown to have little influence on FMD in healthy premenopausal women (35, 36), and participants using these were included in the study.

For imaging, we used Vivid E9 scanner (GE Vingmed Ultrasound, Horten, Norway) with a multiple linear array transducer (6 to 13 MHz). Offline imaging analyses were done with EchoPAC version 203 (GE Vingmed Ultrasound).

The participants were examined in supine position with the right arm resting in unchanged position. The right brachial artery was imaged above the antecubital fossa in the longitudinal plane, and the internal diameter was measured at end-diastole, from the anterior to the posterior endothelial lining of the blood vessel lumen. During image acquisition, anatomic landmarks such as veins, artery branches, and fascial planes were noted, and markers on the skin were drawn to help maintain the exact same image of the artery throughout the study. Occlusion of the brachial artery was done with a blood pressure cuff on the right forearm, inflated at least 50 mmHg above systolic blood pressure.

**Figure 2:**
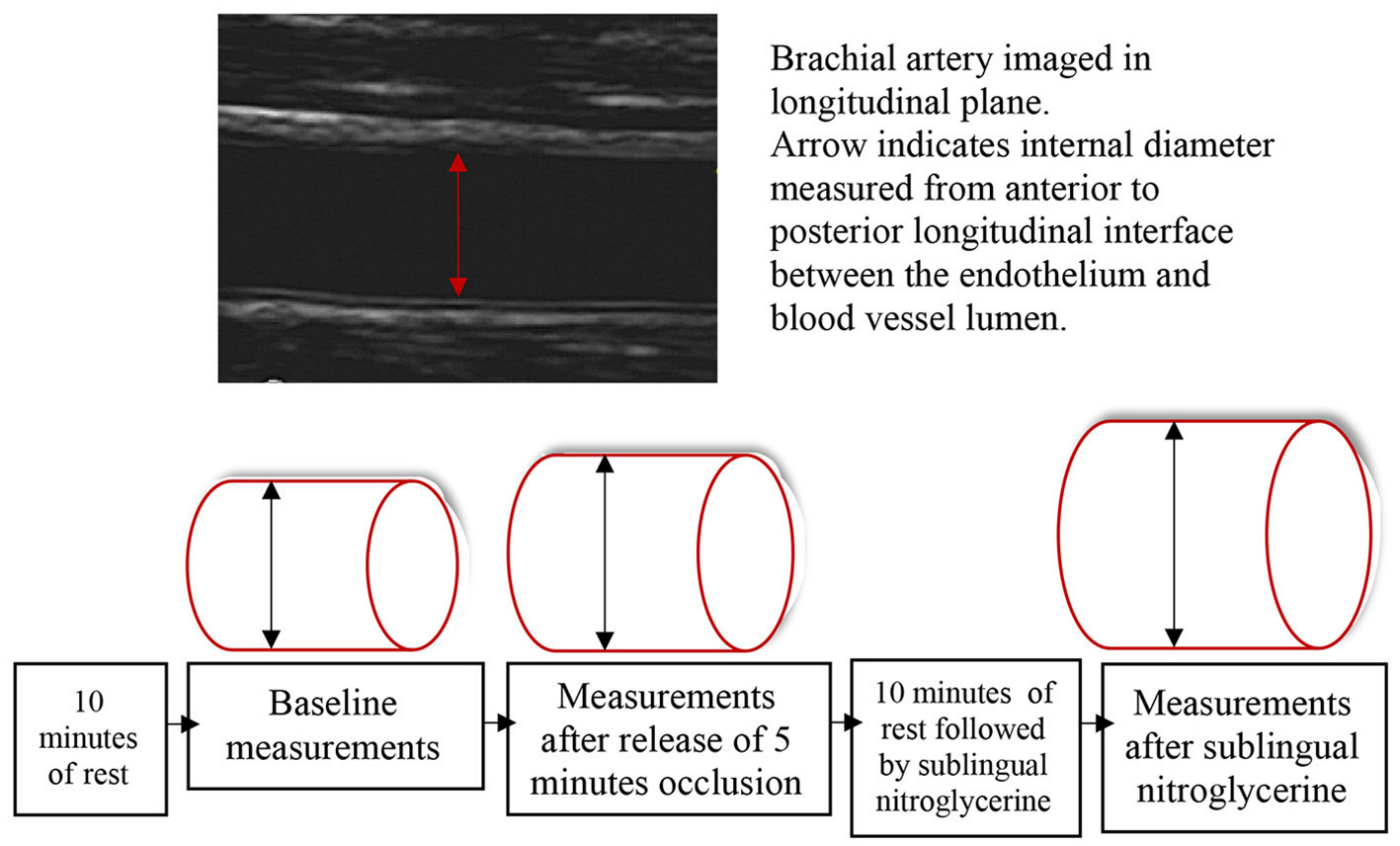
The artery diameter was first measured at baseline after 10 min of rest. Thereafter, occlusion of the right brachial artery was done with a blood pressure cuff on the forearm, inflated at least 50 mmHg above systolic blood pressure for 5 min. The next measurements were done at peak artery dilatation after release of occlusion. After a second 10-min resting period followed by sublingual nitroglycerine administration, the last measurements were done at peak artery dilatation.

Images of the brachial artery were first collected at baseline after 10 min of rest, then repeatedly for 3 min after release of 5-min occlusion, and finally, after a second 10-min resting period, repeatedly for 5 min after a single dose (0.4 mg) of nitroglycerine spray (Nitrolingual; G.Pohl-Boskamp GmbH & Co.KG, Hohenlockstedt, Germany) was administered sublingually. Measurements were done at baseline (Dbaseline), at peak dilatation after release of occlusion (Dflowmediated), and at peak dilatation after sublingual nitroglycerine (Dpeaknitro). For all diameter measurements, an average of three was used. Further details and illustrations of the method are described in the International Brachial Artery Reactivity Task Force (34) and in Figure 2.

FMD was calculated as the absolute diameter change (Dflowmediated – Dbaseline) and the percentage diameter change {[(Dflowmediated – Dbaseline) / Dbaseline] × 100}. The endothelial-independent nitroglycerine-induced dilatation (NID) was calculated as the absolute diameter change (Dpeaknitro – Dbaseline) and the percentage diameter change {[(Dpeaknitro – Dbaseline) / Dbaseline] × 100}. The FMD relative to NID was measured to evaluate the endothelial-dependent dilatation capacity compared with maximal capacity [(Dflowmediated – Dbaseline) / (Dpeaktnitro – Dbaseline) × 100].

A single highly trained sonographer (B.E.) performed all FMD examinations. Another highly trained physician (E.L.) performed control measurements on images of the brachial artery in a random selection of study participant (*n* = 12). The interrater reliability as measured by a two-way mixed-effects model for absolute agreement was 0.99 (95% CI 0.96, 1.00) for Dbaseline measurements, 0.97 (95% CI 0.89, 0.99) for Dflowmediated measurements, and 0.94 (95% CI 0.82, 0.98) for Dpeaknitro measurements.

Information regarding group affiliation (PB/ELBW or control) was unknown to the sonographer at the time of examination.

### Blood Pressure and Blood Sample Measurements

Blood pressure was calculated as the average of three measurements with an automated oscillometric device (Biolight BLT V6; Biolight Meditech Company, Guangdong, China) after 5 min of rest in supine position. Venous blood samples (in ethylenediaminetetraacetic acid–containing tubes) were collected for measurements of HbA1c.

### Statistical Analysis

Gestational age is presented as medians with interquartile ranges (IQRs). Other descriptive variables and outcome data are presented as means with 95% CIs or SD. For comparison of the absolute flow-mediated and nitroglycerine-induced artery diameter changes in PB/ELBW and controls, analysis of covariance (ANCOVA) was used with adjustment for baseline diameter. To examine whether sex, age, BMI, HbA1c level, smoking status, or blood pressure could explain the difference in FMD between PB/ELBW and controls, these variables were also added to the regression model, and an omnibus test on an effect of at least one variable was performed. To examine whether the difference in absolute FMD between PB/ELBW and controls differed by sex, an interaction term for sex and group affiliation was added. For comparison of descriptive variables, percentage flow-mediated and nitroglycerine-induced artery diameter changes from baseline, and endothelial-dependent dilatation capacity (FMD relative to NID) between PB/ELBW and controls, independent samples *t*-tests (with equal variance not assumed) were applied.

Comparison of absolute and percentage FMD between subgroups of PB/ELBW was done with ANCOVA (with adjustment for baseline diameter) and independent *t*-test (with equal variance not assumed), respectively. Comparison of FMD between the three PB/ELBW cohorts was done by Welch's ANOVA. All tests were two-sided, and *p* < 0.05 was considered statistically significant. All statistical analyses were performed using SPSS version 26.0 (IBM Corp., Armonk, NY, USA).

## Results

The characteristics of the study participants are shown in Table 1. The control group was significantly taller than the PB/ELBW group; otherwise, there were no significant differences for age, weight, body mass index (BMI), blood pressure, HbA1c level, or smoking status. In the PB/ELBW and control group, mean BMI was 24.0 (SD 4.2) and 24.4 (SD 4.5) kg/m^2^, mean blood pressure 112/71 (SD 12/9) and 112/69 (SD 11/8) mmHg, and mean HbA1c 32.7 (SD 2.5) and 33.0 (SD 2.6) mmol/mol, respectively. For both groups, mean age was 28 (SD 6) years and 4 (8%) were smokers. The birthweight in the PB/ELBW group ranged from 550 to 1,480 g with mean birthweight 961 (SD 225) g. The GA ranged from 23 to 34 weeks with median GA 27 (IQR 2) weeks. Among the PB/ELBW participants, 14 (28%) were born small for gestational age [<10th percentile according to (37)], 28 (56%) were extremely preterm born (<28 weeks of gestation), 27 (54%) had ELBW (<1,000 g), 19 (38%) had birthweight ≤ 800 g or GA <26 weeks, and 11 (22%) had birthweight ≤ 700 g or GA <25 weeks. Mean baseline diameter of the brachial artery was not significantly different in the control group compared with the PB/ELBW group, 3.37 mm (95% CI 3.19, 3.54) vs. 3.21 mm (95% CI 3.07, 3.35), respectively (*p* = 0.17). None of the participants in the study had history of atherosclerotic cardiovascular disease.

**Table 1:** Characteristics of study participants.

	**PB/ELBW**	**Controls**	***P*-value**
Total, *n* (% males)	50 (48%)	49 (41%)	
Cohort 1 (born 1982–1985), *n*	21	21	
Cohort 2 (born 1991–1992), *n*	16	13	
Cohort 3 (born 1999–2000), *n*	13	15	
Gestational age (weeks), median (IQR)	27 (2)	Term-born	
Birthweight (g), mean (SD)	961 (225)	3,513 (304)	
Small for gestational age, *n*	14 (28%)		
Extremely preterm GA <28 weeks, *n*	28 (56%)		
Extremely low birthweight <1,000 g, *n*	27 (54%)		
Age (years), mean (SD)	28 (6)	28 (6)	0.87
Height (cm), mean (SD)	168.5 (8.2)	172.9 (7.7)	0.01
Weight (kg), mean (SD)	68.4 (14.8)	73.1 (13.5)	0.11
Smokers, n	4 (8%)	4 (8%)	0.98
BMI (kg/m^2^), mean (SD)	24.0 (4.2)	24.4 (4.5)	0.59
HbA1c (mmol/mol) (SD)^a^	32.7 (2.5)	33.0 (2.6)	0.62
Systolic BP (mmHg), mean (SD)	112 (12)	112 (11)	0.86
Diastolic BP (mmHg), mean (SD)	71 (9)	69 (8)	0.38
Baseline diameter^b^ (mm), mean (95% CI)	3.21 (3.07, 3.35)	3.37 (3.19, 3.54)	0.17

*PB/ELBW very preterm born (<29 weeks of gestation) or with extremely low birthweight (<1,000 g). IQR, interquartile range; GA, gestational age; BMI, body mass index; HbA1c, glycosylated hemoglobin; BP, blood pressure*.

a *HbA1c was measured in 78% of the PB/ELBW group and 96% of the control group*.

b *Baseline diameter of the brachial artery*.

### Endothelial-Dependent Flow-Mediated Dilatation (FMD)

**Figure 3:**
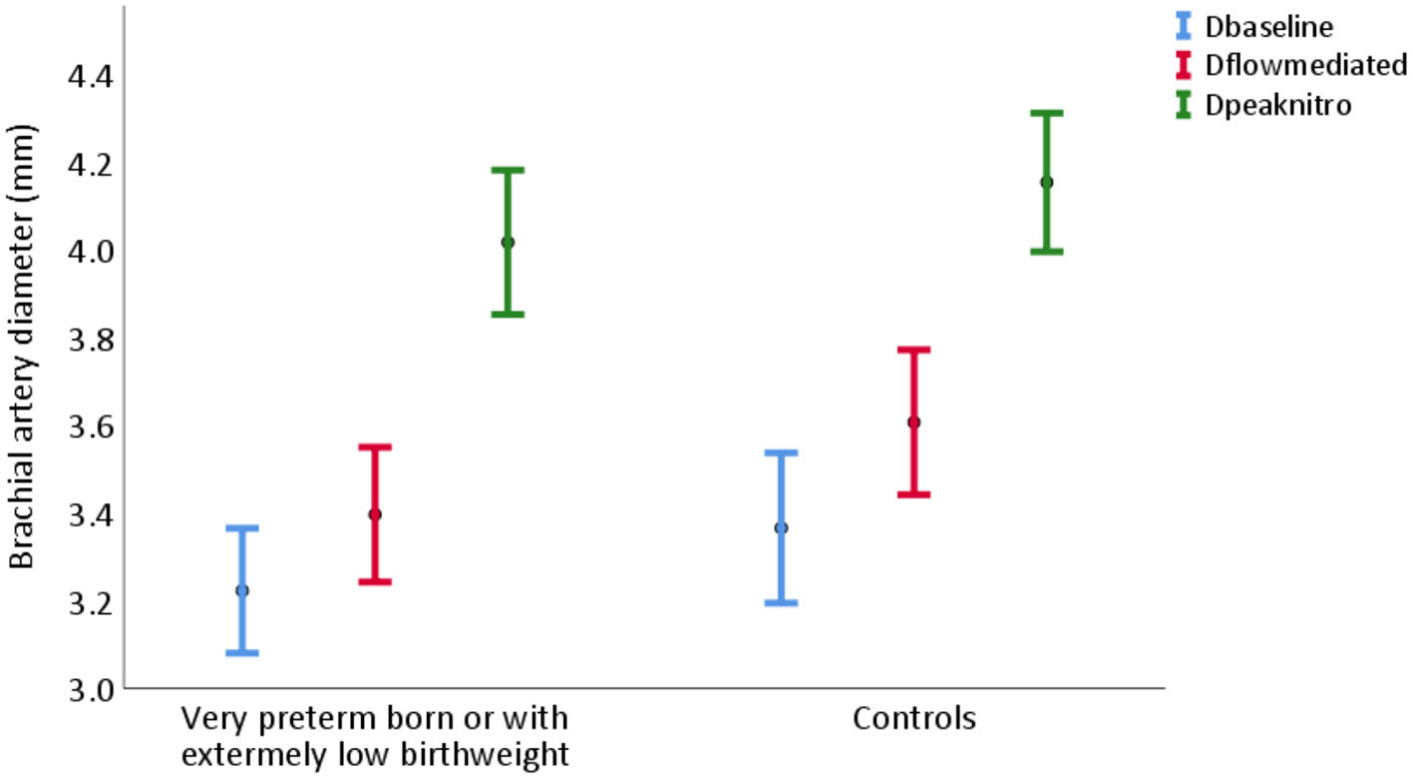
Brachial artery diameter at baseline (blue), after flow-mediated dilatation (red), and after nitroglycerine induced dilatation (green) in young adults born very preterm or with extremely low birthweight (PB/ELBW) and term-born controls, presented as mean with 95% CI. Absolute flow-mediated dilatation was 0.17 mm (95% CI 0.14, 0.21) in the PB/ELBW group and 0.24 mm (95% CI 0.20, 0.28) in the control group. Dbaseline: brachial artery diameter at baseline; Dflowmediated: brachial artery diameter after flow-mediated dilatation; Dpeaknitro: brachial artery diameter after nitroglycerine induced dilatation.

**Figure 4:**
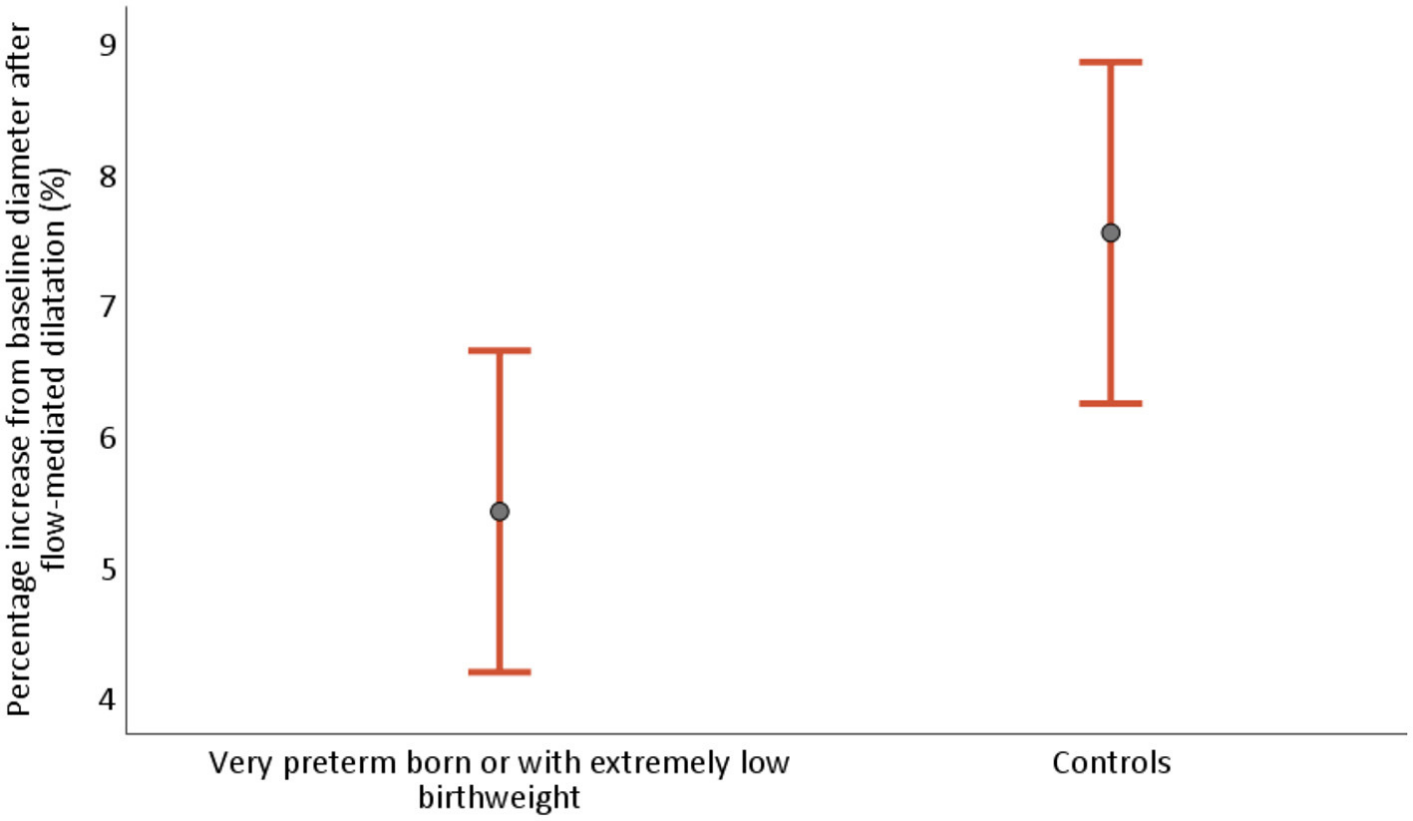
Percentage flow-mediated dilatation (FMD) of the brachial artery from baseline in young adults born very preterm or with extremely low birthweight (PB/ELBW) and term-born controls, presented as mean with 95% CI. Percentage FMD was 5.4% (95% CI 4.2, 6.6) for PB/ELBW and 7.5% (95% CI 6.2, 8.9) for controls.

Table 2 describes measurements of FMD of the brachial artery. The mean absolute FMD was 0.17 mm (95% CI 0.14, 0.21) in the PB/ELBW group vs. 0.24 mm (95% CI 0.20, 0.28) in the control group (*p* = 0.01), and the mean percentage change was 5.4% (95% CI 4.2, 6.6) vs. 7.5% (95% CI 6.2, 8.9), respectively (*p* = 0.02). The absolute brachial artery diameters at baseline, after FMD and after NID, are shown in Figure 3 and the percentage FMD in Figure 4.

**Table 2:** Brachial artery measurements in young adults born very preterm or with extremely low birthweight and term-born controls.

	**PB/ELBW**	**95% CI**	**Controls**	**95% CI**	***P*-value**
	***N* = 50**		***N* = 49**		
Baseline diameter (mm), mean	3.21	(3.07, 3.35)	3.37	(3.19, 3.54)	0.17
Peak diameter at FMD (mm), mean	3.39	(3.24, 3.54)	3.61	(3.44, 3.77)	0.57
Peak diameter at NID (mm), mean	4.02	(3.85, 4.18)	4.15	(4.00, 4.31)	0.23
Absolute FMD (mm), mean^a^	0.17	(0.14, 0.21)	0.24	(0.20, 0.28)	0.01
Absolute NID (mm), mean^a^	0.79	(0.73, 0.86)	0.79	(0.74, 0.83)	1.00
Percentage FMD (%), mean	5.4	(4.2, 6.6)	7.5	(6.2, 8.9)	0.02
Percentage NID (%), mean	25.0	(22.8, 27.2)	24.5	(22.2, 26.8)	0.75
FMD/NID (%), mean	20.3	(16.1, 24.5)	30.8	(25.9, 35.6)	0.001

*PB/ELBW, very preterm born (<29 weeks of gestation) or with extremely low birthweight (<1,000 g); FMD, flow-mediated dilatation; NID, nitroglycerine induced dilatation*.

a *Adjusted for baseline diameter*.

No confounding effect was found for sex, age, BMI, HbA1c level, smoking status, or blood pressure on baseline-adjusted difference in absolute FMD between PB/ELBWs and controls (*p* = 0.56, *n* = 83).

### Nitroglycerine-Induced Dilatation (NID)

There was no significant difference in absolute and percentage NID between the PB/ELBW group and the control group. Mean absolute NID was 0.79 mm (95% CI 0.73, 0.86) in the PB/ELBW group and 0.79 mm (95% CI 0.74, 0.83) in the control group (*p* = 1.0); mean percentage change was 25.0% (95% CI 22.8, 27.2) and 24.5% (95% CI 22.2, 26.8), respectively (*p* = 0.74) (Table 2).

### Endothelial-Dependent Dilatation Capacity (FMD Capacity)

**Figure 5:**
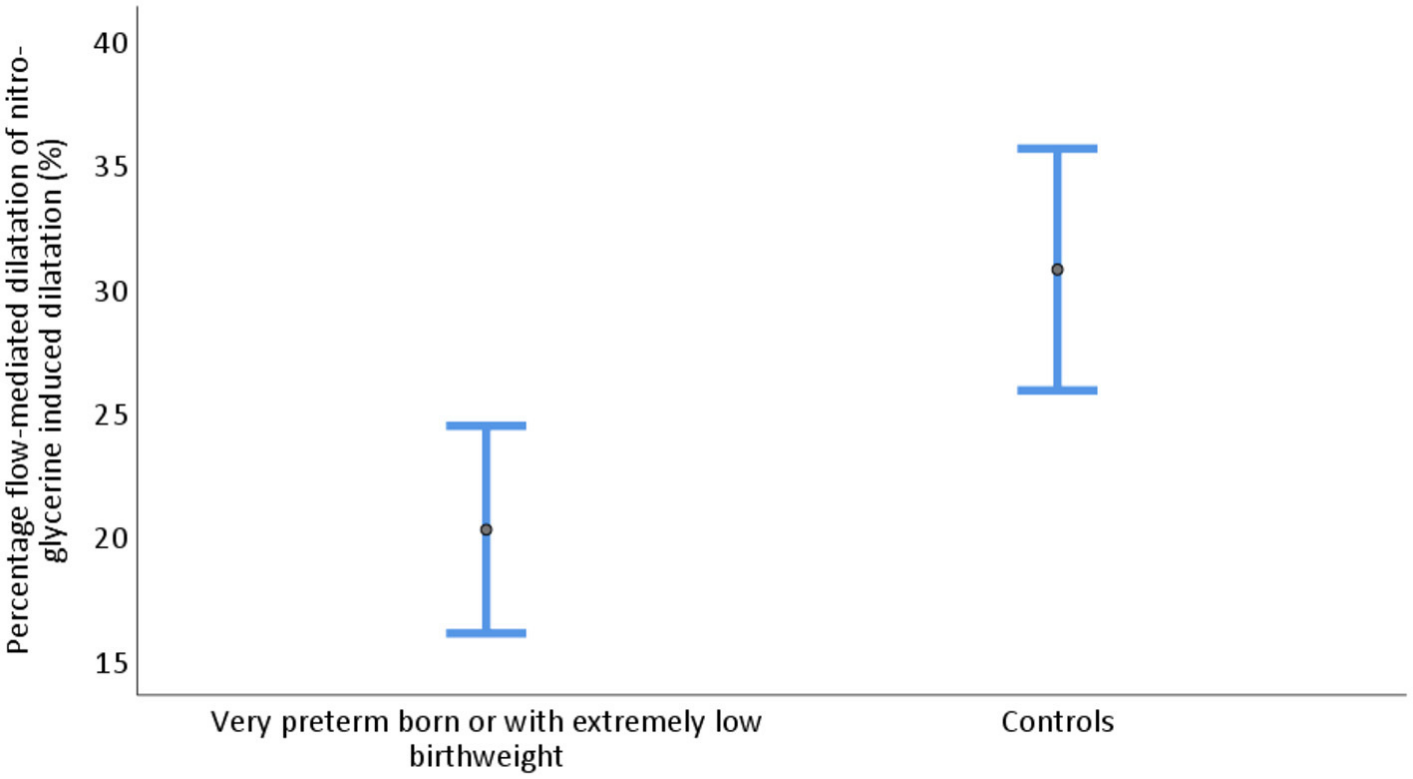
Endothelial-dependent dilatation capacity measured as flow-mediated dilatation in percent of nitroglycerine induced dilatation of the brachial artery in young adults born very preterm or with extremely low birthweight (PB/ELBW) and term-born controls presented as mean with 95% CI. Endothelial-dependent capacity was 20.1% (95% CI 16.1, 24.5) for PB/ELBW and 30.8% (95% CI 25.9, 35.6) for controls.

FMD relative to maximal dilatation capacity measured by NID was significantly lower in the PB/ELBW group compared with the control group. The mean FMD/NID was 20.1% (95% CI 16.1, 24.5) and 30.8% (95% CI 25.9, 35.6), respectively (*p* = 0.001), shown in Table 2 and Figure 5.

### FMD by Cohort and by Subgroup of Birthweight and GA

**Figure 6:**
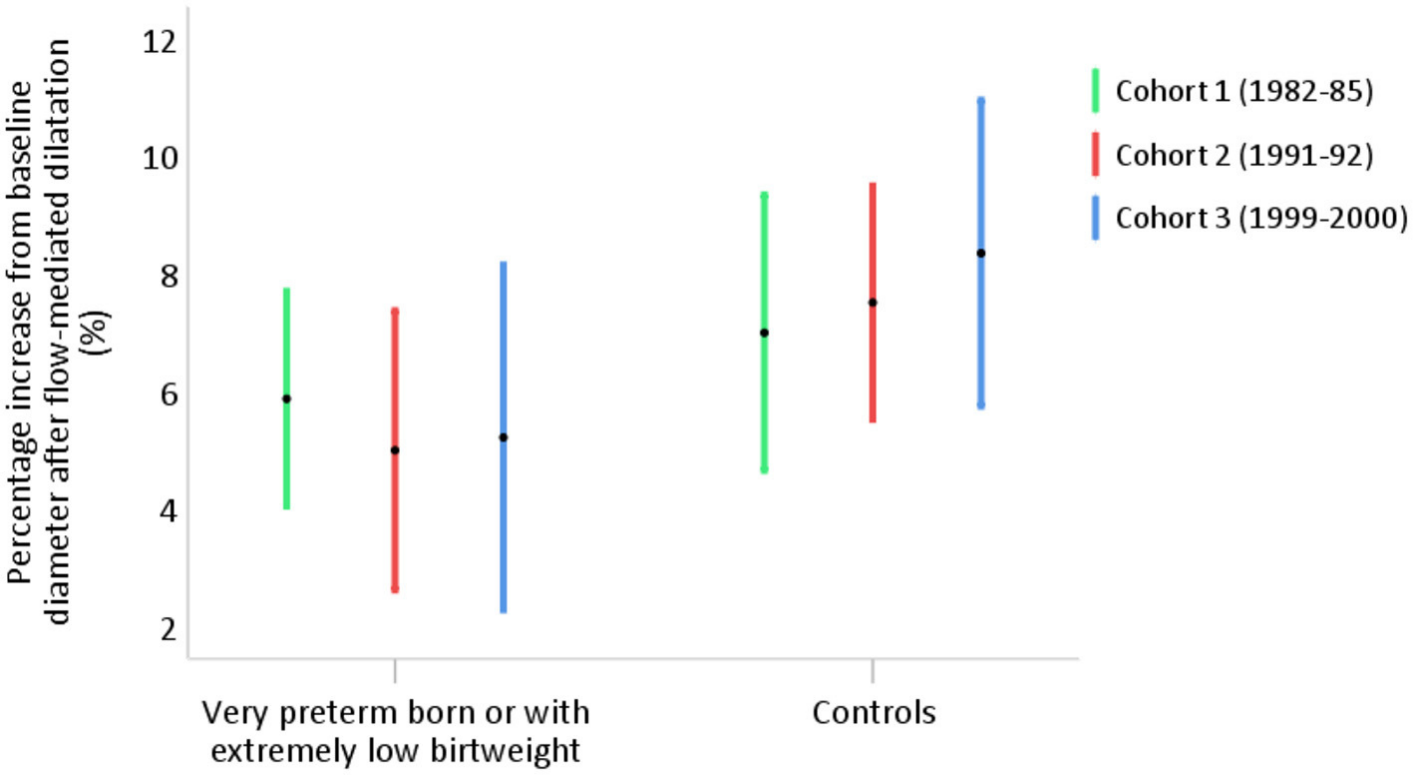
Percentage flow-mediated dilatation of the brachial artery in young adults born very preterm or with extremely low birthweight (PB/ELBW) born in year 1982–1985 (cohort 1, *n* = 21), 1991–1992 (cohort 2, *n* = 16), and 1999–2000 (cohort 3, *n* = 13), presented as mean with 95% CI. Percentage FMD was 5.8% (95% CI 4.0, 7.7) in cohort 1, 5.2% (95% CI 2.6, 7.4) in cohort 2, and 5.2% (95% CI 2.3, 8.2) in cohort 3.

For cohorts 1, 2, and 3, the mean age at assessment was 34 (SD 1.5) years, 27 (SD 0.6) years, and 20 (SD 0.9) years, median GA 28 (IQR 2) weeks, 27 (IQR 3) weeks, and 27 (IQR 1) weeks, and mean birthweight 1,022 (SD 218) g, 942 (SD 238) g, and 885 (SD 208) g, respectively. There was a non-significant tendency of lower FMD in the older cohorts among controls, but we found no difference in absolute or percentage FMD, or FMD capacity, between the three cohorts of PB/ELBW (*p* = 0.6, 0.8, and 0.3, respectively). Characteristics and FMD measures by cohort are shown in Table 3 and Figure 6.

**Table 3:** Characteristics of participants born very preterm or with extremely low birthweight, by cohort.

	**Cohort born 1982–1985**	**Cohort born 1991–1992**	**Cohort born 1999–2000**	***P*-value**
	***n* = 21**	***n* = 16**	***n* = 13**	
Age (years), mean (SD)	34 (1.5)	27 (0.6)	20 (0.9)	<0.01
BW (g), mean (SD)	1,022 (218)	942 (238)	885 (208)	0.2
GA (weeks), median (IQR)	28 (2)	27 (3)	27 (1)	0.3
BMI (kg/m^2^), mean (SD)	24.8 (4.1)	24.6 (3.7)	21.8 (4.5)	0.1
Height (cm), mean (SD)	169.0 (7.5)	169.2 (9.0)	166.7 (8.5)	0.7
Weight (kg), mean (SD)	71.1 (13.7)	70.7 (14.1)	61.2 (16.0)	0.1
Smokers, *n*	3	1	0	0.3
HbA1c (mmol/mol), mean (SD)	32.7 (2.8)	32.5 (2.3)	32.9 (2.3)	1.0
Systolic BP (mmHg), mean (SD)	112 (14)	115 (12)	109 (10)	0.4
Diastolic BP (mmHg), mean (SD)	74 (10)	71 (7)	67 (8)	0.1
Baseline diameter (mm), mean (95% CI)	3.33 (3.14, 3.52)	3.09 (2.18, 3.36)	3.18 (2.40, 3.50)	0.3
Absolute FMD (mm), mean (95% CI)	0.20 (0.14, 0.26)	0.16 (0.08, 0.23)	0.16 (0.08, 0.25)	0.6
Percentage FMD (%), mean (95% CI)	5.8 (4.0, 7.7)	5.0 (2.6, 7.4)	5.2 (2.3, 8.2)	0.8
FMD/NID (%), mean (95% CI)	24.1 (17.1, 31.2)	16.4 (9.7, 23.4)	18.5 (9.4, 27.7)	0.3

*BW, birthweight; GA, gestational age; IQR, interquartile range; BMI, body mass index; HbA1c, glycosylated hemoglobin; BP, blood pressure; FMD, flow-mediated dilatation; NID, nitroglycerine-induced dilatation*.

We also compared subgroups of PB/ELBW with the lowest birthweight and GA with the remaining participants of the PB/ELBW group. We found no significant difference in mean absolute or percentage FMD or FMD capacity between the subgroup with birthweight ≤ 800 g or GA ≤ 26 (38% of PB/ELBW) and the remaining PB/ELBW group (*p* = 1.0, 1.0, and 1.0, respectively), or between the subgroup with birthweight ≤ 700 g or GA ≤ 25 (22% of PB/ELBW) and the remaining PB/ELBW group (*p* = 0.6, 0.7, and 0.3, respectively).

### FMD by Sex

**Figure 7:**
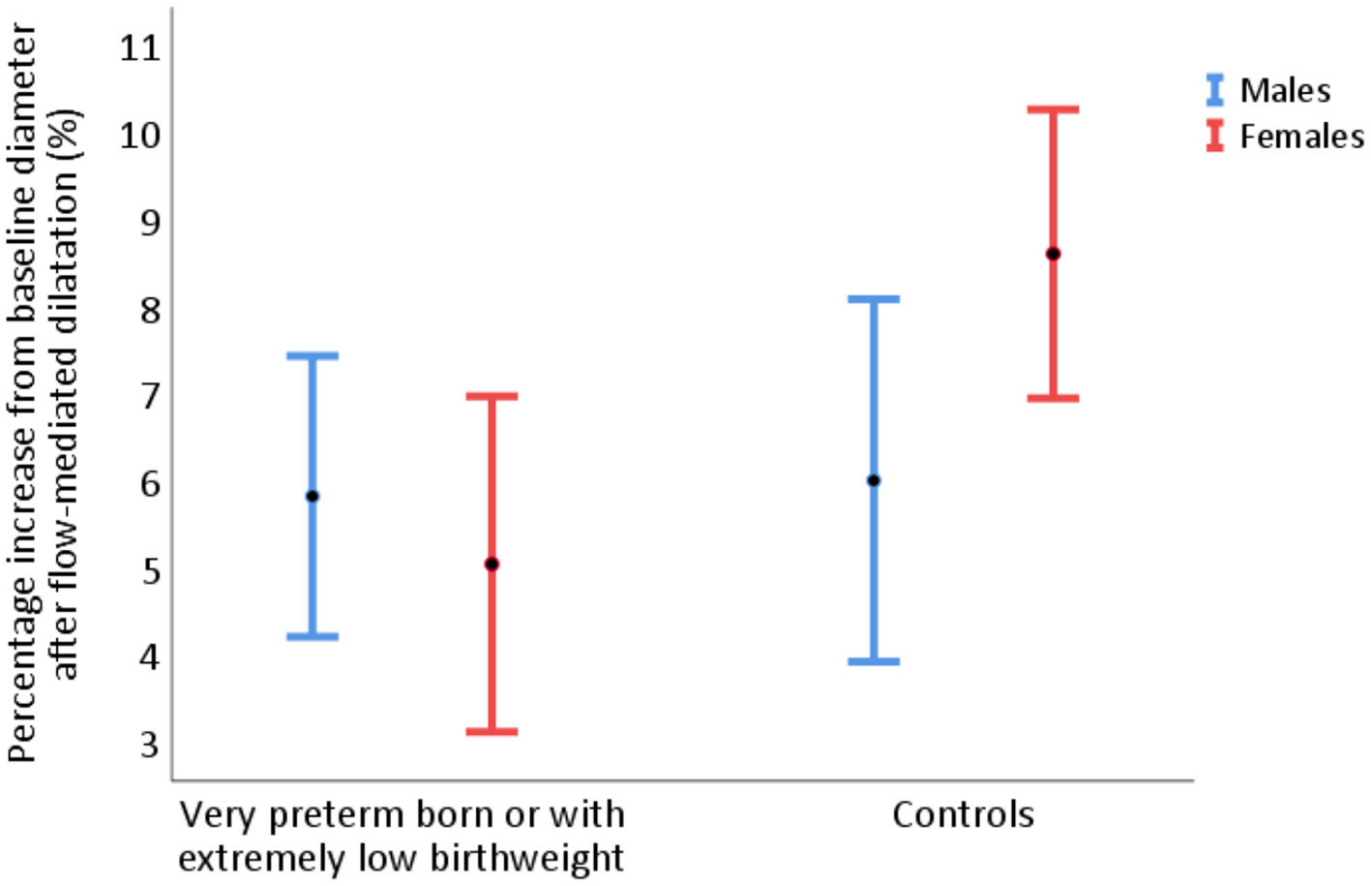
Percentage flow-mediated dilatation (FMD) of the brachial artery in 24 men and 26 women born very preterm or with extremely low birthweight, and term-born controls (20 men and 29 women), presented as mean with 95% CI. In women, percentage FMD was 5.0% (95% CI 3.1, 7.0) for PB/ELBW vs. 8.6% (95% CI 7.0, 10.3) for controls. In men, percentage FMD was 5.8% (95% CI 4.2, 7.4) for PB/ELBW vs. 6.0% (95% CI 3.9, 8.1) for controls.

In analyses of absolute (baseline-adjusted) and percentage FMD separated by sex, we found significantly lower FMD in the female PB/ELBW group compared with the control group, but no significant difference among men. However, the interaction analysis did not show a significant sex effect on the total group difference in absolute FMD (*p* = 0.18). In women, mean absolute FMD was 0.15 mm (95% CI 0.09, 0.20) in the PB/ELBW group vs. 0.25 mm (95% CI 0.21, 0.30) in the control group (*p* = 0.004), with mean percentage change 5.0% (95% CI 3.1, 7.0) vs. 8.6% (95% CI 7.0, 10.3), respectively (*p* = 0.01). In men, mean absolute FMD was 0.20 mm (95% CI 0.15, 0.26) in the PB/ELBW group vs. 0.23 mm (95% CI 0.15, 0.30) in the control group (*p* = 0.3), with mean percentage change 5.8% (95% CI 4.2, 7.4) vs. 6.0% (95% CI 3.9, 8.1), respectively (*p* = 0.9) (shown in Figure 7). The difference in absolute FMD diameter change between PB/ELBW and controls was 0.07 mm (95% CI −0.03, 0.18) greater in the female participants vs. the male participants (p = 0.18).

## Discussion

Among 50 young adults with mean age 28 (SD 6) years born preterm at median GA 27 (IQR 2) weeks, and with mean birthweight 961 (SD 225) g, we found reduced flow-mediated dilatation and endothelial-dependent dilatation capacity of the brachial artery, compared with term-born controls.

Similar to our study, Bassareo et al. (29) found reduced endothelial function measured by finger plethysmography in young adults with mean GA 27.8 (SD 2.2) weeks and mean birthweight 838 (SD 116) g. In addition, Martin et al. (24) found reduced microvascular endothelial function in a small group of school children with mean age 9 (SD 1.4) years and with mean birthweight 2,188 (SD 224) g. Contrary to our findings, Flahault et al. (28), Singhal et al. (26), and Hovi et al. (27) reported normal flow-mediated dilatation of the brachial artery in young adults born preterm or with LBW. Flahault's study included 85 individuals born preterm with mean GA 27.2 (SD 1.4) weeks and birthweight 963 (SD 225) g, similar to our population, whereas the populations in the studies by Singhal and Hovi had higher GA and birthweight [mean GA 31 (SD 2.7) and 29.5 (SD 2.4) weeks, respectively, and mean birthweight 1,400 g (SD 300 g and 112 g, respectively)]. In addition, Shingal's study population was younger with mean age 15 (SD 0.9) years at the time of assessment. Higher GA and birthweight, and younger age at time of assessment, could explain the differences in results in these two studies, compared with the present study.

Previous studies have showed an inverse association between GA and birthweight and the risk of cardiovascular disease (2, 5). We were not able to show a significant difference in FMD or endothelial-dependent dilatation capacity between the subgroups of PB/ELBW with the lowest birthweight and GA, compared with the remaining PB/ELBW group. Similarly, we found no difference in FMD or endothelial-dependent dilatation capacity between the three PB/ELBW cohorts. While the lower mean birthweight in the youngest cohort compared with the oldest cohort (885 g vs. 1,022 g) could lead to a lower FMD, the younger age at the time of assessment and possible improvement in neonatal intensive care treatment the last decades could have the opposite influence on FMD in the younger cohorts. However, the study samples within the subgroups and cohorts of the PB/ELBWs were too small to show minor group differences.

A complex interplay between circulating sex steroid hormones and steroid-independent mechanisms are assumed to contribute to a stated overall difference in endothelial function between healthy young men and women (19). Both sexes show an age-dependent decline in endothelial function (19), and a steeper decline is seen in postmenopausal women (38). This claimed sex difference was, however, doubted by Atkinson et al., who argued that the reported sex differences in fact may be a result of calculation bias because of baseline diameter variations (39, 40). We found a small non-significant difference in mean baseline diameter between the PB/ELBW and control group, and chose to compare the absolute brachial artery diameter changes adjusted for baseline diameter. Fewer men (44%) than women participated in the study, and the sex distribution within each group was slightly unequal. The PB/ELBW group consisted of 48% men and the control group of 41% men. We did, however, not find any sex differences for the main result (absolute FMD in PB/ELBW and controls), although the small sample size provides little statistical power for the interaction analysis.

Most studies assessing FMD of the brachial artery present their results as percentage diameter change from baseline. A cut-off value for FMD indicating normal endothelial function in young adults is not established. The FMD results differ widely between studies with seemingly similar populations, and mean FMD values across populations range from −1.9 to 19.2% (41). Divergent FMD results are partly attributable to methodology and technical aspects of the measurements of the brachial artery. The FMD response may differ with duration, degree, and location of occlusion of the brachial artery (41), with choice of artery, or with method to induce stress to the artery. Possible diurnal variations of FMD may also exist (42, 43). A large Japanese multicenter study proposed a cut-off value for FMD indicating normal vascular function of 8.9% in subjects younger than 40 years and 7.1% independent of age (44). In the present study, the mean FMD in the PB/ELBW group was 5.4%; in the control group, 7.5%. Among the 50 participants in the PB/ELBW group and 49 participants in the control group, 76 and 65% had FMD lower than 8.9% in the respective groups, whereas 66 and 47% had FMD lower than 7.1%.

The interrelationship between endothelial-dependent vasodilatation and endothelial-independent NID and the relation to cardiovascular risk factors and disease are debated (45–48). Impaired NID might reflect vascular smooth muscle cell dysfunction and vascular structure alternations due to atherosclerosis (49, 50). The presence of multiple cardiovascular risk factors or established cardiovascular disease has previously been reported to impair NID of the brachial artery (46).

While a low FMD/NID ratio could indicate cardiovascular risk factors impairing the endothelial function, but no established vascular disease, individuals with established vascular disease and vascular smooth cell dysfunction might have normal or even high FMD/NID ratio despite impaired endothelial function. Assessed together, however, FMD and FMD/NID ratio might give valuable information.

The term of describing endothelial function as endothelial-dependent FMD capacity compared with maximal dilatation capacity, in terms of endothelial-independent NID, is probably most valuable when NID is normal and can be estimated as maximal dilatation. Maruhashi et al. suggested an age-independent cut-off value of 15.6% for NID of the brachial artery in healthy Japanese people (44). We found mean NID of 25% in both the PB/ELBW and control group. Only 8% in the PB/ELBW group and 14% in the control group had NID <15.6%. None of these were smokers, and all had normal values for HbA1c and normal resting blood pressure. Four (one in the PB/ELBW group) were overweight with BMI >25 kg/m^2^. Normal NID response in most of our study participants corresponds well to the population characteristics with a mean age of 28 years, and no established atherosclerotic disease or multiple cardiovascular risk factors. We found decreased endothelial-dependent dilatation capacity in the PB/ELBW compared with controls, 20% vs. 31%, respectively, indicating an impairment of the endothelial function and normal vascular smooth cell function.

The increasing group of adults born very preterm carry a risk of a range of long-term complications and early adult death (2). Although large efforts are invested to facilitate the survival of these infants, the investments in long-term follow-up have been comparatively marginal, and the knowledge on life-long health prospects is sparse. It is especially important to detect modifiable risk factors or early signs of disease in this group, to reduce the life-long disease burden to both individuals and society.

Both indices of microvascular and macrovascular endothelial dysfunction are associated with risk of cardiovascular disease; however, the different methods used to evaluate endothelial function are not interchangeable, and the results may represent different aspects of cardiovascular risk (51). Only a few previous studies have investigated endothelial function in young adults born very preterm and with extremely low birthweight, and to our knowledge, only the study by Flahault et al. (28) investigated macrovascular endothelial function by FMD in this patient group. Our study, therefore, adds important information to the knowledge of the health and future risk of young adults born preterm.

Although dependent on examinator skills, FMD is a preferable method to assess macrovascular endothelial function because it is non-invasive, painless, requires equipment available at any cardiology department, and has a short examination time of about 30 min. It is a well-known method with widespread use in research.

Previous studies have reported an association of traditional cardiovascular risk factors with preterm birth and LBW (6–10), corresponding to an increased risk of cardiovascular disease in the group (2, 18). Our findings of reduced endothelial function in the PB/ELBW group compared with the control group, with no difference in other known risk factors (smoking, BMI, diabetes, hypertension), suggest that extremely low birthweight and very preterm birth might represent individual risk factors for endothelial dysfunction, and thus cardiovascular disease. This association may be due to complex mechanisms triggered already in the intrauterine and neonatal period. Infections and inflammatory processes during pregnancy are major risk factors for premature birth, and might itself give an increased cardiovascular risk (52, 53). Most very preterm born neonates have been exposed to extensive intensive care treatment and long periods of oxygen supplementation that can induce oxidative stress reactions, and might be harmful in a long-term perspective (54). In addition, behavioral issues associated with increased cardiovascular risk, like physical inactivity, may affect cardiovascular health, and previous studies have reported reduced level of activity and physical fitness in preterm born children and adults (55, 56). Maternal smoking or exposure to second-hand smoking during pregnancy (57), and maternal diabetes (58) are associated with preterm delivery and also with risk factors of cardiovascular disease in offspring. There is, however, little evidence that these exposures represent individual risk factors for offspring cardiovascular disease (59, 60). Further studies are needed to investigate the causal pathways of the associations between preterm birth or low birthweight and cardiovascular risk.

The study was part of a regional population-based cohort study of individuals born PB/ELBW and individually age- and sex-matched term-born controls recruited to long-term follow-up since year 2000. The study design, and similarity of the PB/ELBW and control group regarding characteristics and cardiovascular risk factors at the time of the assessment reduce the risk of confounding. The method of measuring endothelial function is well-established (20, 61). To diminish procedure-related variance, the measurements were performed by a highly trained single sonographer with strict adherence to the International Brachial Artery Reactivity Task Force, and the calculated interrater variability was excellent (62).

There were limitations to this study. Only 34% of the eligible PB/ELBW subjects were examined. The PB/ELBWs who did not participate had similar median GA and mean days of ventilatory support and oxygen demand, compared with those who participated (Table 4). The subjects who did not participate had a slight and non-significant lower mean birthweight (896 g vs. 961 g, *p* = 0.08), and a higher occurrence of moderate or severe bronchopulmonary dysplasia (63) compared with the PB/ELBWs who participated. Flahault et al. described an association between bronchopulmonary dysplasia and altered elastic properties of the brachial artery, but found no similar association with impaired FMD (28). The possible selection bias could, however, most likely lead to an underestimation of the difference in FMD between the PB/ELBW and the control group. Although the PB/ELBW and control group were well-matched for several known cardiovascular risk factors, we had no information regarding cholesterol levels or family history of cardiovascular disease. Previous studies have found no association between maternal familial hypercholesterolemia and preterm birth or LBW (64), but a possible association of preterm birth with dyslipidemia (65). A small risk of unknown confounding cannot be ruled out. However, as all study participants were normotensive and all measurements of HbA1c were well beneath the cut-off for prediabetes, and the BMI was similar in the two groups, there is in our opinion not a high risk of lipid discrepancy between the groups. The measurements of HbA1c were incomplete. However, the BMI was normal in the 13 study participants with missing HbA1c (mean BMI was 21.8 kg/m^2^), indicating a low risk of metabolic syndrome in these subjects.

**Table 4:** Characteristics of participants and non-participants born very preterm or with extremely low birthweight.

	**Participants (*n* = 50)**	**Non-participants (*n* = 98)**	***P*-value**
Male/female, n	24/26	50/48	0.08
Cohort 1/2/3, n	21/16/18	28/19/51	
BW, g, mean (SD)	961 (225)	896 (178)	
GA, weeks, median (IQR)	27 (2)	27 (2)	0.3
Days on ventilator, mean (SD)	8 (10)	8 (9)	0.9
Days on CPAP, mean (SD)	28 (14)	31 (19)	0.6
Days on O_2_ supplement, mean (SD)	51 (37)	57 (42)	0.4
Moderate/severe BPD, *n*	28%	45%	0.04
Prenatal steroid treatment, *n*	48%	60%	0.3
Postnatal steroid treatment, *n*	18%	25%	0.3
Surfactant, *n*	40%	52%	0.2

*Cohort 1, born 1982–1985; cohort 2, born 1991–1992; cohort 3, born 1999–2020; BW, birthweight; GA, gestational age; IQR, interquartile range; CPAP, continuous positive airway pressure; O_2_, oxygen; BPD, bronchopulmonary dysplasia (classified as moderate/severe BPD if need for supplemental O_2_ or CPAP at 36 weeks of gestation)*.

In conclusion, the young adults born very preterm (<29 weeks of gestation) or with extremely low birthweight (<1,000 g) had lower flow-mediated vasodilatation and reduced endothelial-dependent dilatation capacity of the brachial artery compared with term-born controls. Reduced FMD is suggestive of vascular endothelial dysfunction, and well-established as an early marker for development of atherosclerotic cardiovascular disease. Our findings suggest that very preterm birth and extremely low birthweight may be independent risk factors for cardiovascular disease. We therefore recommend follow-up programs for these children to extend into adulthood, with special attention to reduce modifiable risk factors for atherosclerotic cardiovascular disease, and increase awareness of early symptoms of cardiovascular disease in adults born very preterm.

## Data Availability Statement

The raw data supporting the conclusions of this article will be made available by the authors, without undue reservation.

## Ethics Statement

The studies involving human participants were reviewed and approved by the Regional Committee for Medical and Health Research Ethics of the Western Norway Health Authority (REC 2017/0068). The patients/participants provided their written informed consent to participate in this study.

## Author Contributions

BE conceived and designed the analysis, collected and organized data, carried out the analyses, drafted the initial article, and revised the article. ME coordinated data collection, organized data, and critically reviewed the article for important intellectual content. GG conceptualized and designed the study and critically reviewed the article for important intellectual content. MV provided funding, designed the data collection instruments, coordinated and supervised data collection, and critically reviewed the article for important intellectual content. KH gave advice on the analysis of data, has participated in the interpretation of data, and has critically reviewed the article for important intellectual content. EL conceptualized and designed the study and critically reviewed the article for important intellectual content. All authors approved the final article and take responsibility for all aspects of reliability of the data presented and their discussed interpretation.

## Funding

Western Norway Health Authority covered expenses running the long-term follow-up in Project Extreme Prematurity (assignment number 912011). Additional funding was assigned from Norwegian Association for Children with Heart Disease (project number 123) and Bergen Heart Foundation (project number 101708).

## Conflict of Interest

The authors declare that the research was conducted in the absence of any commercial or financial relationships that could be construed as a potential conflict of interest.

## Publisher's Note

All claims expressed in this article are solely those of the authors and do not necessarily represent those of their affiliated organizations, or those of the publisher, the editors and the reviewers. Any product that may be evaluated in this article, or claim that may be made by its manufacturer, is not guaranteed or endorsed by the publisher.
