# Supplementary material for: Vascular Endothelial Function Assessed by Flow-Mediated Vasodilatation in Young Adults Born Very Preterm or With Extremely Low Birthweight: A Regional Cohort Study
Source: Front Pediatr. 2021 Sep 24;9:734082. doi: 10.3389/fped.2021.734082 (PMC8500064; doi:10.3389/fped.2021.734082)
Supplement: Supplementary file 1 [file Table_1.DOCX]

**Supplementary table 1: Definitions of preterm birth and birthweight**

| Extremely preterm born | Gestational age <28 weeks |
| --- | --- |
| Very preterm born | Gestational age 28-32 weeks |
| Moderate preterm born | Gestational age 32-34 weeks |
| Late preterm born | Gestational age 34-37 weeks |
| Term born | Gestational age ≥37 weeks |
| Extremely low birthweight | <1000 g |
| Very low birthweight | 1000 g-1500 g |
| Low birthweight | 1500 g-2500 g |
| Normal birthweight | ≥2500 g |
